# Retrospectively assessed psychosocial working conditions as predictors of prospectively assessed sickness absence and disability pension among older workers

**DOI:** 10.1186/s12889-018-5047-z

**Published:** 2018-01-17

**Authors:** Emil Sundstrup, Åse Marie Hansen, Erik Lykke Mortensen, Otto Melchior Poulsen, Thomas Clausen, Reiner Rugulies, Anne Møller, Lars L. Andersen

**Affiliations:** 1 National Research Centre for the Working Environment, Lersø Parkallé 105, 2100 Copenhagen, Denmark; 2 Department of Public Health, University of Copenhagen, Copenhagen, Denmark; 3 Center for Healthy Aging, University of Copenhagen, Copenhagen, Denmark; 4 Department of Psychology, University of Copenhagen, Copenhagen, Denmark; 5 Department of Occupational Medicine, Copenhagen University Hospital Holbæk, Copenhagen, Denmark; 6 The Research Unit for General Practice and Section of General Practice, Department of Public Health, University of Copenhagen, Copenhagen, Denmark; 7 Department of Health Science and Technology, Physical Activity and Human Performance group, SMI, Aalborg University, Aalborg, Denmark

**Keywords:** Disability pension, Early retirement, Sickness absence, Influence at work, Appreciation, Social support, Psychosocial demands, Psychosocial work characteristics

## Abstract

**Background:**

The aim was to explore the association between retrospectively assessed psychosocial working conditions during working life and prospectively assessed risk of sickness absence and disability pension among older workers.

**Methods:**

The prospective risk of register-based long-term sickness absence (LTSA) and disability pension was estimated from exposure to 12 different psychosocial work characteristics during working life among 5076 older workers from the CAMB cohort (Copenhagen Aging and Midlife Biobank). Analyses were censored for competing events and adjusted for age, gender, physical work environment, lifestyle, education, and prior LTSA.

**Results:**

LTSA was predicted by high levels of cognitive demands (HR 1.31 (95% CI 1.10–1.56)), high levels of emotional demands (HR 1.26 (95% CI 1.07–1.48)), low levels of influence at work (HR 1.30 (95% CI 1.03–1.64)), and high levels of role conflicts (HR 1.34 (95% CI 1.09–1.65)). Disability pension was predicted by low levels of influence at work (HR 2.73 (95% CI 1.49–5.00)) and low levels of recognition from management (HR 2.04 (95% CI 1.14–3.67)).

**Conclusions:**

This exploratory study found that retrospectively assessed high cognitive demands, high and medium emotional demands, low influence at work, low recognition from management, medium role clarity, and high role conflicts predicted LTSA and/or disability pension.

## Background

The majority of high-income countries are facing a rapidly ageing population challenging economies and welfare systems under pressure. A long, healthy and productive working life is, therefore, a political priority and longer working lives are expected in the future [1]. To meet this objective, statutory retirement age is increasing in many European countries in parallel with reduced possibilities for early retirement benefits. However, longer working careers may increase the risk of suboptimal health and therefore challenge work participation at an older age. For instance, the working-lives of older employees are often characterized by a long history of different psychosocial work characteristics, all of which may contribute to health status in older age. Knowledge on risk factors for health-related adverse labor market outcomes, such as long-term sickness absence (LTSA) and disability pension, are therefore needed to secure a sustainable workforce.

Previous studies have suggested that adverse psychosocial working conditions increase the risk of ill health and sickness absence [2–7]. Specifically, the combination of high psychosocial demands and low control at work (i.e. Karasek’s job strain model [8]) has been extensively studied in the literature, and has been shown to be associated with sickness absence [5, 9–12]. In a prospective cohort study among British civil service employees (the Whitehall II study), Head and et al. [4] showed that adverse changes in decision latitude and job demands were associated with increased risk of LTSA, and that improvements in social support at work were associated with reduced risk of LTSA. This was further confirmed by Lidwall et al. [5], who showed that high strain jobs and lack of social support increased the odds for LTSA. In addition, Clausen et al. [3] observed that lack of job resources such as influence at work, are more important predictors of LTSA than high job demands among 39,408 Danish employees. Another Danish study reported that LTSA was predicted by high cognitive and emotional demands and by role conflicts [13].

Psychosocial working conditions have also been shown to impact premature exit from the labor market demonstrated by studies showing that low job control [14, 15] and low skill discretion [16] are predictive of future disability pension. A study from the English Longitudinal Study of Ageing found that adverse psychosocial work factors (i.e. higher effort-reward imbalance and lower job control) predicted premature exit from the labor market [17]. In addition, Virtanen et al. showed that high work time control was associated with extended employment into older age among 4677 older Finnish employees [18] and Leijten and co-workers observed that higher autonomy, higher support, and lower psychosocial job demands reduced the risk of disability pension among workers with health problems by 82%, 49%, and 11%, respectively [19]. In a recent systematic review and meta-analysis, Knardahl et al. [20] found moderate evidence for the role of low job control, and for the combination of high demands and low control (job strain) as predictors for disability pension, and recommended the measurement of specific exposure factors in future studies.

The vast majority of studies examining the contribution of the psychosocial work environment to the risk of LTSA and disability pension are limited to the job strain model and seem to have disregarded other psychosocial working conditions. Further, in most studies, exposure ascertainment is usually limited to the exposure present at baseline and disregard exposures at earlier points in the working life. Thus, there is a need for studies considering other factors than job strain and studies that try to examine psychosocial factors over a longer period of time [20]. Poor health is an established risk factor for leaving the labor market and disability pension seems to be preceded by sickness absence. Exploring whether and to what extent numerous psychosocial work environment factors are associated with these health-related labor market outcomes could shed additional light on the complex interaction of health and work characteristics underlying the occurrence of premature labor market exit. All psychosocial work characteristics can be thought of as potentially hazardous, however, their association with disability pension (permanently unable to work due to ill health) and LTSA (temporarily unable to work due to ill health) need further investigation.

The present study aims to address the limitations of the existing literature by exploring the prospective association of 12 psychosocial work environment factors with risk of LTSA and disability pension. The psychosocial factors include both components of the job strain model (quantitative demands, work pace, influence at work and possibilities for development) as well as numerous other psychosocial work environment factors (emotional demands, cognitive demands, social community at work, social support from colleagues, social support from supervisors, role conflicts, role clarity and recognition from management). Further, participants were asked at baseline not to report their current exposure but to assess, retrospectively, their whole work life exposure to these psychosocial work environment factors.

The study has an exploratory approach rather than a theory-testing approach. Thus, a specific, theory-based hypothesis about the association of a specific psychosocial exposure with LTSA and disability pension was not formulated. Instead, the study explores whether and to what extent the 12 psychosocial work environment factors are associated with one or both outcomes. The approach has been documented in a study protocol that was published before data analyses commenced [21].

## Methods

### Study design

This prospective follow-up study links data on work environment and health from the Copenhagen Aging and Midlife Biobank (CAMB) with a Danish register containing information on labor market attachment. The design and methods of the study are described elsewhere [21]. To ensure transparent and standardized reporting of the study, the STROBE checklist was followed [22].

### Study population

CAMB contains data on biological, psychological and social factors for persons between 49 and 63 years of age from the merging of three established cohorts: The Metropolit Cohort [23], The Copenhagen Perinatal Cohort [24] and the Danish Longitudinal Study on Work, Unemployment, and Health [25]. Of the 17,937 individuals invited for the CAMB data collection in 2009–2011, 7190 responded to the questionnaire and 5575 attended a clinical examination. More information on the study population can be found elsewhere [26]. For the present study, employed wage earners at baseline (*n* = 5076) were included. Because not all participants answered all the survey questions, the exact number of individuals for each analysis varies. Baseline characteristics of the study population are illustrated in Table 1. The present manuscript is part of a larger study setup investigating the influence of physical and psychosocial work environment throughout life and physical and cognitive capacity in midlife on labor market attachment among older workers. From this larger study setup, a study protocol and analyses on the physical work environment have previously been published [21, 27, 28].

**Table 1:** Characteristics of the study population

	N	Percent	Mean	SD
Age, years	5076		54.3	3.8
Gender				
Men	3537	70		
Women	1539	30		
Education				
Unskilled	366	7		
Skilled	1869	38		
Short-education	509	10		
Medium-education	1330	27		
Long-education	902	18		
Lifestyle				
BMI (kg/m^2^)	5076		26.0	4.1
Physical activity (1–4; high-low)	5076		2.7	0.65
Smoking (yes and ex-smoker)	1102	22		
Smoking (no)	3922	78		
Physical work environment during working life				
Sedentary work	2618	53		
Moderate physical work	1072	22		
Hard physical work	827	17		
Very hard physical work	414	8		
Chronic diseases				
Back disease (have or have had)	1306	26		
No back disease	3705	74		
Cancer inclusive leukemia (have or have had)	212	4		
No cancer inclusive leukemia	4799	96		
Chronic depression or anxiety (have or have had)	516	10		
No chronic depression or anxiety	4497	90		

Values are percentage of participants or mean and SDs. *BMI* body mass index

### Psychosocial work characteristics

Psychosocial working conditions throughout working life were assessed retrospectively by 12 items on specific psychosocial work characteristics derived and modified from the COPSOQ [29]. Each item represents one distinct COPSOQ scale. The original COPSOQ scales were of multiple items (with the exception of work pace that is a single item COPSOQ scale), but due to limited space, it was decided in the CAMB study to only include one item per scale.. The items and their associated response categories can be seen in Table 2. They belong to three COPSOQ domains: *Demands at work* (quantitative demands, work pace, cognitive demands, emotional demands), *Work organization and job contents* (influence at work, possibilities for development), and *Interpersonal relations and leadership* (recognition from management, role clarity, role conflicts, social support from colleagues, social support from supervisors, social community at work) [29]. For the purpose of analysis, the five response categories of each of the 12 psychosocial work characteristics were reduced to three categories (high, medium, low) with the high category consisting of the two response categories in strongest agreement with the item, the medium category corresponding to the middle response category, and the low category corresponding to the two response categories in least agreement with the item [3].

**Table 2:** Psychosocial working conditions ascertained in CAMB

Scale	Items	Response categories
Domain of demands at work:		
Quantitative demands	How often did you not have time to complete all your work tasks?	type 1
Work pace	Did you have to work very fast?	type 1
Cognitive demands	Did your work require you to make difficult decisions?	type 1
Emotional demands	Did you have to relate to other people’s personal problems as part of your work?	type 1
Domain of work organisation and job content:		
Influence	Did you have a large degree of influence concerning your work?	type 1
Possibilities for development	Did you have the possibility of learning new things through your work?	type 2
Domain of interpersonal relations and leadership:		
Recognition from management	Was your work recognized and appreciated by the management?	type 2
Role clarity	Did you know exactly which areas were your responsibilities?	type 2
Role conflicts	Were contradictory demands placed on you at work?	type 2
Social support from colleagues	Did your colleagues talk with you about how well you carry out your work?	type 1
Social support from supervisors^1^	Did your nearest superior talk with you about how well you carry out your work?	type 1
Social community at work	Was there a good atmosphere between you and your colleagues?	type 1

Response category type 1: always; often; sometimes; seldom; never/hardly never

Response category type 2: to a very large extent; to a large extent; somewhat; to a small extent; to a very small extent

In addition, supplementary post-hoc analyses combining the 12 psychosocial work environment factors into higher-ordered constructs were performed. This included: (i) three COPSOQ domains of demands at work, work organization, and job content, and interpersonal relations and leadership, (ii) a demand - job control ratio, and (iii) a demand - reward ratio. The demand – job control ratio was created by dividing the demand domain by the domain of work organization and job content to approximate the job strain model [30]. The demand-reward ratio was created by dividing the demand domain by score combining the items of “recognition from management”, “social support from co-workers” and “social support from supervisors” to approximate the effort-reward imbalance model [31].

### LTSA and disability pension

Information on LTSA and disability pension was derived from the Danish Register for Evaluation of Marginalization (DREAM), containing information on all social transfer payments in Denmark [32] and linked to the CAMB cohort via the unique Danish personal identification number. In DREAM, sickness absence is recorded on a weekly basis when the employer is entitled to reimbursement of the sickness pay [33]. In Denmark, the employers have the right to receive sickness compensation benefits from the municipalities for an employee experiencing sickness absence for a given minimum period (up to 2007,> 13 days; 2007–2008,> 14 days; since 2008,> 21 days; since 2012 > 30 days) [32]. Hence, during our follow-up, the period during which the employer received no reimbursement for employee sickness absence changed from 21 days to 30 days of sickness absence (January 2012). To define LTSA consistently throughout this period, it was defined as sickness absence > 30 calendar days, corresponding to ≥6 consecutive weeks in DREAM. In Denmark, a disability pension is a social benefit for people with a significant and permanent loss of work ability with a compensation period lasting until retirement age. Individuals with a permanent loss of work ability and working on special terms, such as “light jobs” (work on special terms with a wage subsidy offered to people on disability pension) and “flexible jobs” (a job offer on special terms for people with permanently reduced work ability), or vacancy benefit for individuals with flexible job, were also classified as receiving disability pension [34].

### Covariates

Physical work environment throughout working life was assessed by the following question: “Looking back on your entire working life: For how many years of your working life have you had…, 1) mostly sedentary work without physical strain?, 2) mostly standing or walking work without major physical strain?, 3) mostly standing or walking work with some lifting and carrying?, 4) mostly heavy, fast or physically demanding work?”. For each response category respondents listed the number of years of working life (cumulative exposure assessment) with the specific effort level [35]. The data on exposure years in each of the 4 categories was transformed to a number between 0 and 100, where 0 indicates that all exposure years belong to category 1 (sedentary work) and 100 indicates that all exposure years belong to category 4 (very hard physical work), and anything in between was linearly scaled. The categories were defined as “low physical work demands” (0–24.99), “moderate physical work demands” (25–49.99), “high physical work demands” (50–74.99) and “very high physical work demands” (75–100) [27].

Physical activity level during leisure time was assessed by the following question: “What would you say best describes your spare time physical activities?” with the following response categories: 1) go for competitive sport regularly and several times a week; 2) go for physical training or heavy house or garden work at least 4 h per week; 3) go for walks, biking or other kinds of light exercise at least 4 h a week (including Sunday excursions, lighter garden work and biking/walking to and from work); 4) read, watch television or have other sedentary activities. For further analyses, a variable was generated with the number ranging from 1 to 4 representing the selected response category.

Chronic diseases were assessed by the following question: “Do you have or have you had any of the following diseases?” with the response options “yes, have now”, “yes, previously” or “no” to the following diseases: back disease, cancer including leukemia, chronic anxiety or depression. Three diseases were included - back disease, cancer including leukemia, and chronic anxiety or depression - because these were the only diseases that predicted LTSA in a previous analysis of Danish employees [36]. For each chronic disease, a binary variable was generated: 1) representing that the participant has or have had the specific disease, and 2) representing that the participant never had the specific disease.

Level of education was categorized into five groups; unskilled, skilled, and short-, medium-, and long education [37]. Skilled labor refers to labor that requires workers who have specialized training or a learned skill-set to perform the work. These workers could be either blue-collar or white-collar workers, with varying levels of training or education. Unskilled labor refers to labor that requires no other education or professional qualifications than primary school education. Short-, medium-, and long education refer to short-, medium-, or long-cycle further education than a high-school education. For further analyses, a variable was generated corresponding to the educational level, with 1 representing the lowest level of education (i.e. unskilled) and 5 representing the highest level of education (i.e. long education).

Smoking was evaluated by a question from the CAMB questionnaire: “Do you smoke?” With the response categories: 1. Yes, daily; 2. Yes, but not daily; 3. No, but I have smoked previously; 4. No, I have never smoked. For the present analyses, response category 1, 2 and 3 were collapsed into “smoking” while response category 4 represented “never smoking”.

Height and weight of participants were measured by the clinical personal and body mass index (BMI) was calculated as BMI (kg/m^2^) = body weight/(height)^2^.

### Statistical methods

The Cox proportional hazard model [38, 39], censoring for competing events, was used for modeling the risk of register-based LTSA and disability pension during the 4–6 year follow-up period (i.e. baseline measurements were collected from 2009 to 2011 and register follow-up took place in 2015). A competing risk approach was employed censoring for all events of permanent drop-out from the labor market within the follow-up period. For instance, the analysis with disability pension as outcome was censored for statutory retirement, early retirement, immigration, and death (derived from DREAM). The participant was censored in the sense that nothing was observed or known about the participant after the time of censoring. A censored participant may or may not have an event after the end of observation time. On the contrary, when individuals had an onset of LTSA or disability pension within the follow-up period, the survival times were non-censored and referred to as event times. Analyses were carried out separately for each individual psychosocial work characteristic and adjusted for multiple confounders: age, gender, physical work environment, lifestyle factors (physical activity level, BMI, smoking), chronic diseases, education, and LTSA over the preceding two years prior to baseline (derived from the DREAM register). The estimation method was maximum likelihood and the results are reported as hazard ratios (HR) with 95% confidence intervals (CI).

In the supplementary post-hoc analyses, the 12 single psychosocial work environment factors were replaced with five higher-ordered constructs, including the three COPSOQ domains of demands at work, work organization and job content and interpersonal relations and leadership, a demand-job control ratio and a demand-reward ratio. The standardized Cronbach’s alpha of these new constructs were 0.57 (COPSOQ demands domain), 0.48 (COPSOQ work organization and job content domain, which was identical with job control score), and 0.65 (COPSOQ interpersonal relations and leadership domain), respectively.

## Results

### Onset of LTSA and disability pension

During the follow-up period, the following number of outcome events occurred: 970 individuals (19.3%) had at least one episode of LTSA and 85 individuals (1.7%) received disability pension.

### Psychosocial work characteristics as predictors of LTSA and disability pension

Table 3 shows the result for the association of psychosocial work characteristics with risk of LTSA and disability pension. LTSA was predicted by high levels of cognitive demands (HR 1.31 (95% CI 1.10–1.56), *p* = 0.003), high levels (HR 1.26 (95% CI 1.07–1.48), *p* = 0.005) and medium levels of emotional demands (HR 1.19 (95% CI 1.02–1.40), *p* = 0.031), low levels of influence at work (HR 1.30 (95% CI 1.03–1.64), *p* = 0.028), medium levels of role clarity (HR 1.33 (95% CI 1.11–1.59), *p* = 0.002) and high levels of role conflicts (HR 1.34 (95% CI 1.09–1.65), *p* = 0.007).

**Table 3:** The association of the 12 psychosocial work characteristics throughout working life with risk of LTSA and disability pensioning

				LTSA HR		Disability pension	
		N	Percent	(95% CI)	*P*-value	HR (95% CI)	*P*-value
Quantitative demands	Low	2328	46.9	1		1	
	Medium	1603	32.3	0.91 (0.78–1.05)	0.199	1.09 (0.65–1.82)	0.751
	High	1037	20.9	1.15 (0.97–1.36)	0.101	0.87 (0.46–1.65)	0.670
Work pace	Low	563	11.3	1		1	
	Medium	2225	44.7	0.93 (0.74–1.16)	0.526	0.93 (0.45–1.95)	0.852
	High	2188	44.0	0.99 (0.79–1.23)	0.914	0.64 (0.29–1.39)	0.257
Cognitive demands	Low	1587	32.0	1		1	
	Medium	2046	41.2	1.14 (0.97–1.33)	0.110	0.68 (0.40–1.18)	0.170
	High	1331	26.8	1.31 (1.10–1.56)	0.003	0.97 (0.53–1.76)	0.908
Emotional demands	Low	1971	39.7	1		1	
	Medium	1477	29.7	1.19 (1.02–1.40)	0.031	0.90 (0.49–1.63)	0.896
	High	1523	30.6	1.26 (1.07–1.48)	0.005	1.59 (0.92–2.77)	0.098
Influence at work	High	3722	74.8	1		1	
	Medium	930	18.7	1.15 (0.98–1.35)	0.079	1.67 (0.98–2.84)	0.057
	Low	321	6.5	1.30 (1.03–1.64)	0.028	2.73 (1.49–5.00)	0.001
Posibilities for development	High	3981	80.1	1		1	
	Medium	863	17.4	1.05 (0.89–1.24)	0.567	0.80 (0.45–1.43)	0.445
	Low	129	2.6	0.85 (0.59–1.22)	0.375	2.05 (0.94–4.48)	0.071
Recognition from management	High	2814	57.3	1		1	
	Medium	1649	33.6	1.05 (0.91–1.21)	0.472	1.19 (0.70–2.02)	0.514
	Low	452	9.2	1.18 (0.95–1.46)	0.128	2.04 (1.14–3.67)	0.017
Role clarity	High	4299	86.4	1		1	
	Medium	624	12.6	1.33 (1.11–1.59)	0.002	1.71 (0.97–3.01)	0.066
	Low	51	1.0	0.58 (0.29–1.13)	0.109	0.60 (0.08–5.51)	0.620
Role conflicts	Low	2790	56.4	1		1	
	Medium	1639	33.1	1.15 (1.00–1.33)	0.050	1.15 (0.70–1.89)	0.574
	High	517	10.5	1.34 (1.09–1.65)	0.007	0.98 (0.45–2.12)	0.958
Social support from colleagues	High	1320	26.9	1		1	
	Medium	2138	43.5	0.97 (0.82–1.13)	0.659	0.85 (0.48–1.50)	0.570
	Low	1456	29.6	0.95 (0.80–1.12)	0.526	0.99 (0.55–1.77)	0.972
Social support from supervisors	High	1304	26.2	1		1	
	Medium	2440	49.1	0.88 (0.75–1.02)	0.097	0.81 (0.46–1.41)	0.452
	Low	1227	24.7	0.86 (0.72–1.03)	0.110	1.29 (0.72–2.32)	0.388
Social community	High	4735	95.2	1		1	
	Medium	223	4.5	1.08 (0.83–1.40)	0.566	1.56 (0.74–3.30)	0.248
	Low	18	0.4	1.85 (0.92–3.76)	0.087	3.55 (0.83–15.27)	0.088

Adjusted for age, gender, physical work environment, lifestyle, chronic diseases, socioeconomic position, previous LTSA

*HR* hazard ratio, *95 CI* 95% confidence intervals

Disability pension was predicted by low levels of influence at work (HR 2.73 (95% CI 1.49–5.00), *p* = 0.001) and low levels of recognition from management (HR 2.04 (95% CI 1.14–3.67), *p* = 0.017).

### Post-hoc analyses on higher-ordered constructs as predictors of LTSA and disability pension

Table 4 shows the result of the post-hoc analyses on the three COPSOQ domains, and on the demand-job control ratio and the demand-reward ratio. LTSA was predicted by high demands, poor work organization and job content and medium demand-reward ratio. Disability pension was predicted by medium and poor work organization and job content.

**Table 4:** The association of three COPSOQ domains, demand-job control ratio and demand-reward ratio throughout working life with risk of LTSA and disability pensioning

			LTSA		Disability pension	
	N	Percent	HR (95% CI)	*P*-value	HR (95% CI)	*P*-value
Domain of demand at work						
Low	1637	33	1		1	
Medium	1429	29	1.13 (0.95–1.33)	0.165	0.83 (0.46–1.48)	0.526
High	1913	38	1.19 (1.02–1.40)	0.031	0.86 (0.49–1.50)	0.595
Domain of work organization and job content						
Good	1700	34	1		1	
Medium	1738	35	1.11 (0.94–1.32)	0.216	2.93 (1.33–6.44)	0.008
Poor	1541	31	1.26 (1.06–1.49)	0.008	3.18 (1.46–6.90)	0.003
Domain of interpersonal relations and leadership						
Good	1880	38	1		1	
Medium	1364	27	1.21 (1.02–1.42)	0.025	0.89 (0.46–1.70)	0.715
Poor	1736	35	1.12 (0.96–1.31)	0.141	1.48 (0.88–2.48)	0.140
Demand-job control ratio						
Low	1648	33	1		1	
Medium	1628	33	1.12 (0.96–1.13)	0.148	0.78 (0.46–1.34)	0.372
High	1703	34	0.96 (0.81–1.13)	0.148	0.57 (0.31–1.08)	0.085
Demand-reward ratio						
Low	1646	33	1		1	
Medium	1647	33	1.18 (1.00–1.38)	0.041	0.93 (0.548–1.59)	0.803
High	1682	34	1.15 (0.98–1.35)	0.095	0.76 (0.43–1.35)	0.348

Adjusted for age, gender, physical work environment, lifestyle, chronic diseases, socioeconomic position, previous LTSA

*HR* hazard ratio, *95 CI* 95% confidence intervals

## Discussion

Of the 12 psychosocial work environment factors, high cognitive demands, high and medium emotional demands, low influence at work, low recognition from management, medium role clarity, and high role conflicts predicted LTSA and/or disability pension. In the post-hoc analyses of higher-ordered psychosocial constructs, the COPSOQ domains of high demands, medium and poor work organization and job content, and medium interpersonal relations and leadership, as well as a medium demand-reward ratio, predicted LTSA and/or disability pension.

The study found that some of the psychosocial work characteristics affected LTSA and disability pension differently. The only psychosocial work characteristic that was related to both outcomes were influence at work. It is in agreement with previous research that low influence at work predicted the risk of disability pension, showing the importance of influence at work in participants’ latest job function for future disability pension [14, 15, 17, 20]. In a recent systematic review, Knardahl et al. [20] found that low level of job control was consistently associated with disability pension and therefore concluded that moderate evidence exists for the effect of this psychosocial work characteristic on disability pension. In the present study, the analyses were adjusted for education, but residual confounding may still exist. Therefore, an exploratory analysis adjusted for social class (classified by occupation and coded into Social Classes I-VIII, according to the standards of the Danish Occupational Social Class classification [40]) was performed (not shown in the tables). This did not change the results - low influence still predicted the risk of disability pension.

Demands at work (i.e. cognitive and emotional demands) predicted LTSA but not disability pension. Several studies have reported associations between psychological, emotional and cognitive demands at work and risk of sickness absence [4, 7, 13]. In line with the present results, previous studies found no association between job demands and work exit [18, 41, 42], which was supported by Fleischmann et al. (2017) who did not found evidence that low job demands in midlife protected against retirement or health-related labor market exit among middle-aged (35–55) men and women from the Whitehall II study [43]. Hence it seems that a high level of demands at work, and in particular emotional demands, influence the transition into LTSA, whereas other aspects of the psychosocial working environment are more important for permanently leaving the labour market due to poor health (i.e. disability pension).

Within the domain of work organization and job content, no associations between possibilities for development and any of the outcomes was found. In contrast, several studies using multi-item assessment methods have previously reported an association between skill discretion and labour market exit [16, 43–45]. Specifically, Lund et al. (2005) found that low skill discretion significantly increased the risk of voluntary early retirement pension among 365 older employees (57–62 years) in Denmark [45]. In the present study, possibilities for development was measured by a single-item from the COPSOQ questionnaire (regarding the possibility of learning new things through work), whereas skill discretion in the above studies was assessed by multi-item scales (e.g. 6 items form the Karasek’s Job Content Questionnaire [43]), which could have reduced comparability. A more thorough discussion of the limitations of the single-item approach can be seen in the strengths and limitations section below.

Within the domain of interpersonal relations and leadership, social support from managers and colleagues was not associated with any of the outcomes whereas a low level of recognition by the management predicted the risk of disability pension, but not LTSA. There seem to be mixed results in the literature in regard to the relation between low social support and labour market exit [43]. Carr et al. (2016) found that low social support was not statistically associated with increased odds of work exit in longitudinal data on 3462 workers aged 50–69 from five waves of the English Longitudinal Study of Ageing (ELSA) [42]. In contrast, Fleischmann et al. [43] and Lund et al. [45] showed that social support was associated with premature exit from the labour market, in middle-aged (35–55 years) and older workers (57–62 years), respectively.

Previous studies have observed that low recognition is predictive of both early retirement [42, 44] and sickness absence [46, 47]. In the present study, low recognition by the managers predicted the risk of disability pension but was not associated with LTSA. Hence, being appreciated and recognized during an occupational career seems to reduce the risk of leaving the labour market due to ill health (i.e. disability pension). Overall, the present results suggest that the associations with psychosocial working conditions during working life might affect LTSA and disability pension differently, even though they both represent health-related labor market outcomes.

When examining combinations of the 12 single-items, high demands at work predicted LTSA, whereas poor work organization and job content predicted both LTSA and disability pension. The COPSOQ domain of work organization and job content in the present study consisted of the scales “influence at work” (which is similar to “decision authority” in the job strain model) and “possibilities for development” (which is similar to “skill discretion” in the job strain model). However, when building a proxy for job strain by combining the demand dimension with the work organization and job content dimension, this new measure neither predicted LTSA nor disability pension. This is in disagreement with literature reviews that suggest job strain to be an important predictor for both LTSA and disability pension [12, 20, 48]. It is unknown whether this is explained by the limitations of the proxy measure or by other reasons.

### Strengths and limitations

It was examined whether numerous psychosocial work environment factors, measured with single items, predicted the risk of LTSA or disability pension. The study was motivated by reviews showing that the vast majority of psychosocial work environment studies have examined job strain as a risk factor for LTSA and disability pension while failing to study other potentially important psychosocial work environment factors [12, 20, 48]. Thus, the study was explorative, i.e. the aim of the study was not to test a specific hypothesis, but to explore a wide range of psychosocial exposures.

Psychosocial working conditions during working life were retrospectively assessed by self-reports at mid-life and could, therefore, be prone to potential bias, in particular, recall bias. Thus, it was not possible to analyze the psychosocial work environment throughout the whole working life, as the psychosocial working conditions were only measured once when he participants were asked to assess retrospectively how these conditions were during their working career. It seems reasonable to assume that psychosocial working conditions are not static during the course of a working life, but instead are prone to changes. Asking participants to combine exposures during their working life in a single number for their average level of influence at work, social relations or role conflicts (among others) is only a first and very limited step towards a life course perspective in occupational psychosocial epidemiology. At the next step, studies are needed examining changing psychosocial working conditions repeatedly over time and whether exposure is more important at specific time points and less important at other time points. In addition, the retrospective assessment of earlier working conditions in our study may be affected by current health- and psychological status [49, 50], although this bias was probably reduced by the adjustment for baseline chronic diseases.

Another limitation to the study is that all psychosocial constructs were measured with single-items from the COPSOQ questionnaire and not with multi-item scales that have undergone a thorough psychometric testing. Not using the entire validated scales hampers the comparison to other studies measuring psychosocial working conditions by all multi-item scales from the COPSOQ. Due to competition in space in the CAMB questionnaire survey, a high number of scales with single items were preferred over a low number of scales with multiple items. The single-item approach allowed for the exploration of a wide range of different exposures, responding to requests in the literature to broaden the perspective on psychosocial risk factors of sickness absence and disability pension [20]. However, the result of the post-hoc analyses on the three COPSOQ domains exhibited a similar pattern of results as the analyses based on single-items, which lends credence to the findings in the main analysis of the present study.

The low response rate of the CAMB survey is a limitation to the study and could have led to selection bias. Previous analyses of the CAMB cohort found that participants and non-participants were comparable in terms of health and education but that a greater proportion of participants than non-participants were employed at the time of the survey suggesting that the study sample may represent a partly socially selected group [26]. In addition, it cannot be ruled out that those with the longest history of hazardous psychosocial exposures could already be outside the labor market when the present study was initiated, suggesting that the healthy worker effect could be a source of confounding bias.

The low number of cases for disability pension is an important limitation to the study. Thus, a possible explanation for the lack of association between most psychosocial factors and risk of disability pension could have been insufficient statistical power. As two predictor variables and two endpoints were included, the number of statistical tests conducted was high and it cannot be ruled out that some of the statistically significant associations might have occurred by chance. Whether or not analyses should be adjusted for multiple testing is controversially discussed in the literature [51, 52]. After weighing the arguments it was decided not to adjust for multiple testing, also because of the exploratory nature of the study.

Caution is warranted regarding the generalization of the results to countries other than Denmark or the Scandinavian countries. Van der Wel et al. [53] previously stated that the welfare systems of Scandinavian countries are better at protecting against non-employment due to illness than other systems. Lunau et al. [54] reported that the association of adverse psychosocial working conditions and risk of depressive disorders was weaker in countries with active labour market policies, high unemployment benefits and low-income inequality (e.g. Denmark) and stronger in countries that lack these labour and social policies. Hence, future studies should examine the generalizability of the present results to other countries with different arrangements of disability pensions and labor market protection.

A strength of the study is the use of data on labor market outcomes derived from the DREAM register that has high reliability, because all transfer payments are systematically recorded [55]. Hence, DREAM makes it possible to assess labor market attachment that is free from potential bias from self-reported LTSA and disability pension. Additionally, by using register data on labor market attachment, common methods variance (i.e. associations between exposure and outcome due to same assessment methods) and recall bias with regard to outcome ascertainment was eliminated [56]. A somewhat high prevalence of LTSA was observed in the present study, where 19.3% of the study population had at least one episode of this outcome event during the follow-up period. Pre-study power calculations, based on extracts from the DREAM database of people aged 50–59 years who were working at baseline (*n* = 757,226), revealed an incidence rate of 11.7% (new cases of LTSA) over a 3 year period (from 2009 to 2011) [21]. Taken into account the longer follow-up time (3-5 years), this is somewhat in agreement with the proportion that we observed in the present study sample. In addition, the introduction of a new disability pension reform in 2012 have made it more difficult to be granted a disability pension in Denmark and the average age to obtain a disability pension has increased from 45.8 years in 2011 to 48.1 years in 2015. Thus, it can be suggested that a proportion of people between 50 and 60 years with declining health are not anymore applicable for a disability pension, which could cause an increased probability of sickness absence. In line with this, we observed a lower incidence of disability pension in the study population compared with pre-study power calculations performed before the introduction of the new disability pension reform [21].

## Conclusions

This exploratory study found that retrospectively assessed high cognitive demands, high and medium emotional demands, low influence at work, low recognition from management, medium role clarity, and high role conflicts predicted LTSA and/or disability pension.

## Abbreviations

CAMB: Copenhagen Aging and Midlife Biobank
COPSOQ: Copenhagen Psychosocial Questionnaire
DREAM: Danish Register for Evaluation of Marginalization
LTSA: Long-term sickness absence
